# Impact of Donor Age on Short‐Term Outcomes After Pediatric Split Liver Transplantation

**DOI:** 10.1111/petr.70444

**Published:** 2026-08-27

**Authors:** Dongwei Wu, Jianfeng Zhang, Kaining Zeng, Xiao Feng, Qing Yang, Jia Yao, Binsheng Fu, Yang Yang, Hui Tang, Shuhong Yi

**Affiliations:** ^1^ Organ Transplantation Center The Third Affiliated Hospital of Sun Yat‐sen University Guangzhou China

**Keywords:** donor age, graft survival, pediatric split liver transplantation, portal vein thrombosis, short‐term outcomes

## Abstract

**Background:**

Split liver transplantation (SLT) expands access to grafts for pediatric recipients, but the short‐term implications of donor age at the extremes of the accepted range remain uncertain.

**Methods:**

We retrospectively analyzed 144 pediatric SLT recipients treated from 2015 to 2025. Recipients were grouped by donor age: 2–10 years (*n* = 21), 11–49 years (*n* = 103), and 50–63 years (*n* = 20). One‐year patient and death‐censored graft survival were assessed using Kaplan–Meier methods. Donor age was additionally modeled continuously per 10‐year increase in separate Cox models for death‐censored graft loss and patient death. The functional form of donor age for graft loss was examined using restricted cubic splines, and outcomes were explored by in situ versus ex situ splitting.

**Results:**

One‐year patient survival was 90.5%, 91.2%, and 84.4% in Groups A, B, and C, respectively (log‐rank *p* = 0.711); corresponding death‐censored graft survival was 95.2%, 94.0%, and 84.1% (*p* = 0.334). Per 10‐year increase in donor age, the hazard ratio was 1.217 (95% CI, 0.823–1.800; *p* = 0.324) for graft loss and 1.099 (95% CI, 0.796–1.516; *p* = 0.568) for patient death. No significant departure from linearity was detected (*p* for nonlinearity = 0.527). In situ and ex situ splitting showed no significant differences in 1‐year survival after adjustment for donor age.

**Conclusions:**

One‐year survival did not differ significantly by donor age. Carefully selected young and older donors may be considered in experienced centers, although limited sample sizes and wide confidence intervals warrant caution.

## Graphical abstract

In a single‐center cohort of 144 pediatric split liver transplant recipients, 1‐year patient survival and death‐censored graft survival did not differ significantly among donor age groups of 2–10, 11–49, and 50–63 years. Donor age was also not significantly associated with graft loss or patient death when analyzed continuously. Carefully selected young and older donors may be considered in experienced centers, although small extreme‐age groups and wide confidence intervals warrant caution.

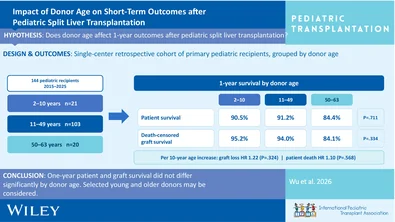

**Abbreviations**
ALT: alanine aminotransferase
AST: aspartate aminotransferase
CI: confidence interval
DBD: donation after brain death
DCD: donation after circulatory death
GRWR: graft‐to‐recipient weight ratio
HAT: hepatic artery thrombosis
HR: hazard ratio
ICU: intensive care unit
INR: international normalized ratio
IQR: interquartile range
LT: liver transplantation
MELD: model for end‐stage liver disease
PELD: pediatric end‐stage liver disease
PNF: primary nonfunction
PVT: portal vein thrombosis
SLT: split liver transplantation
TBIL: total bilirubin

## 1 Introduction

Pediatric liver transplantation is an effective treatment for end‐stage liver disease [1, 2], but the shortage of suitable donor livers remains critical. Split liver transplantation (SLT) can expand the donor pool and reduce waiting‐list mortality [3, 4, 5]. Although SLT was initially associated with high complication and mortality rates [6, 7], advances in surgical technique and donor–recipient matching have substantially improved outcomes [1, 8, 9, 10].

The effect of donor age on pediatric SLT remains uncertain. Donors aged 10 years or younger and those aged 50 years or older have historically been regarded as higher risk or outside conventional donor‐selection standards for splitting. For young donors, small vessel caliber may increase the risk of vascular complications [11, 12]. For older donors, many centers use an upper age limit of approximately 40–50 years [9, 13, 14, 15, 16, 17]. We therefore prespecified thresholds at 10 and 50 years. The labels used in this report also show the observed minimum and maximum donor ages: 2–10 years in Group A, 11–49 years in Group B, and 50–63 years in Group C.

This study compared 1‐year outcomes after pediatric SLT across these donor‐age groups and evaluated donor age as a continuous exposure. We also explored whether the splitting technique influenced perioperative recovery, complications, or 1‐year survival.

## 2 Methods

### 2.1 Study Design and Participants

This retrospective single‐center cohort included patients younger than 18 years who underwent primary SLT at the Third Affiliated Hospital of Sun Yat‐sen University between January 2015 and December 2025. Patients who underwent whole‐liver transplantation, living‐donor liver transplantation, repeat SLT, or had missing core data on donor age, 1‐year survival, or major complications were excluded. A total of 144 recipients from 124 donors were included. Twenty donors provided split grafts to two recipients each.

**FIGURE 1:**
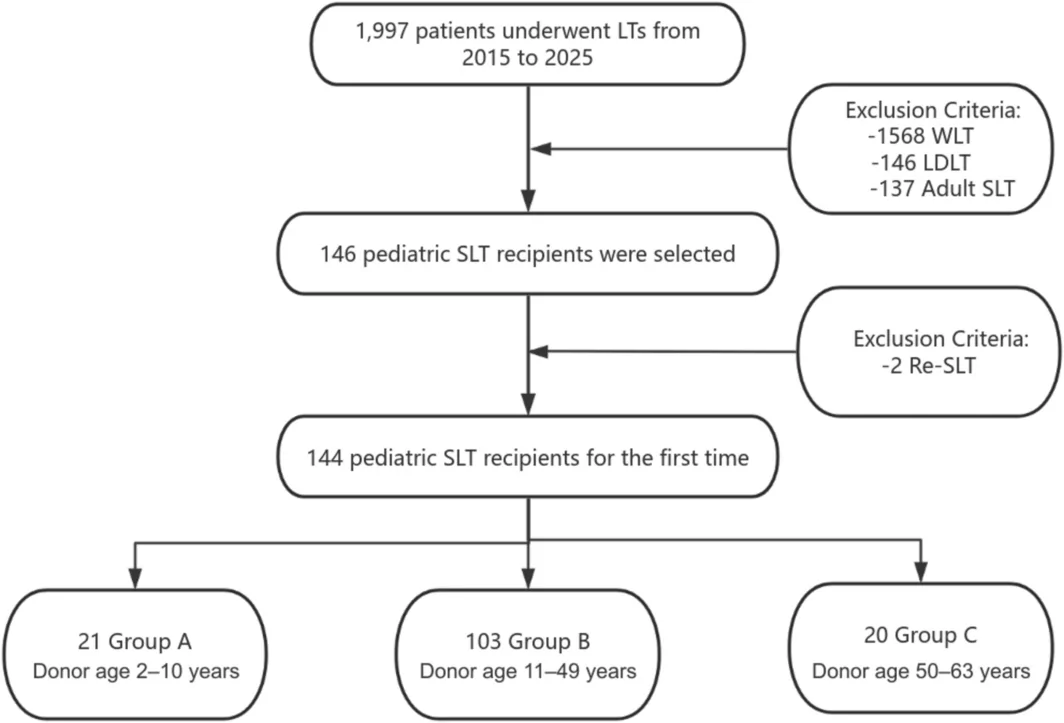
Study flow diagram. Age‐group labels report the observed donor‐age ranges. LDLT, living‐donor liver transplantation; SLT, split liver transplantation; WLT, whole‐liver transplantation.

Donors were categorized using prespecified clinical thresholds: Group A, ≤ 10 years (observed range 2–10 years; *n* = 21 recipients); Group B, 11–49 years (observed range 11–49 years; *n* = 103); and Group C, ≥ 50 years (observed range 50–63 years; *n* = 20) (Figure 1). These thresholds were chosen because donors aged ≤ 10 years and ≥ 50 years have historically been considered higher risk or outside standard SLT donor‐selection criteria [11, 12, 13, 14, 15, 16, 17].

The study conformed to the Declaration of Helsinki and the Declaration of Istanbul. Donor organs were obtained through voluntary donation after brain death or circulatory death with family consent. No organs were obtained from executed prisoners or through unethical procurement. The Institutional Ethics Committee of the Third Affiliated Hospital of Sun Yat‐sen University approved the study (No. II2026‐120‐01).

### 2.2 Surgical Technique

In situ splitting was defined as completion of hepatic parenchymal transection before graft reperfusion and cold preservation; ex situ splitting was performed on the back table after organ perfusion [18]. For donors after brain death (DBD) and donors after circulatory death (DCD) with hemodynamic stability, in situ splitting was preferred, predominantly using the classic split into a left lateral segment (segments II and III) and an extended right lobe (right trisegment graft, segments I and IV–VIII). In young donors (2–10 years), the celiac trunk/left hepatic artery was allocated to the left lateral segment, while the main portal vein and the main bile duct were allocated to the extended right lobe. Alternatively, a complete left lobe/right lobe split with division of the middle hepatic vein could be performed, with the left and right hemilivers reconstructed using iliac vein grafts and arterial patches, respectively. The side retaining the main bile duct was kept consistent with the side retaining the main hepatic artery to avoid compromising the biliary blood supply. In grafts from low‐body‐weight donors, the main hepatic artery was preferentially retained with the left graft at the surgeon's discretion. Volume reduction was performed according to the graft‐to‐recipient weight ratio (GRWR); when GRWR exceeded 4%, marginal volume reduction was carried out with preservation of the main trunk of the middle hepatic vein. Cholangiography was routinely performed before and after splitting to delineate the biliary anatomy. All recipients underwent piggyback liver transplantation. Portal vein reconstruction was performed by end‐to‐end anastomosis (using continuous or interrupted sutures). The hepatic artery was anastomosed under an operating microscope with 8‐0 or 9‐0 non‐absorbable sutures. Biliary reconstruction was achieved by Roux‐en‐Y hepaticojejunostomy.

### 2.3 Donor Selection Criteria

All potential donors for pediatric SLT at our center were evaluated using a single uniform set of selection criteria, regardless of age. The criteria were as follows: Basic donor requirements: body mass index ≤ 26 kg/m^2^; no overt systemic infection; intensive care unit stay ≤ 5 days; hemodynamically stable; no history of cardiopulmonary resuscitation or cardiac arrest; minimal use of high‐dose vasopressors; ABO blood type compatibility with the intended recipient; and serum sodium ≤ 160 mmol/L. Pre‐procurement functional assessment: no significant liver dysfunction, defined as alanine aminotransferase (ALT) and aspartate aminotransferase (AST) ≤ 3 times the upper limit of normal (ULN), total bilirubin (TBIL) ≤ 2 × ULN; no evidence of hepatic fibrosis; and absent or only mild microvesicular steatosis (< 10%). Intra‐procurement and anatomical evaluation: estimated cold ischemia time ≤ 10 h; smooth liver texture with uniform perfusion; imaging (computed tomography or magnetic resonance cholangiopancreatography) confirming no major vascular or biliary anomalies that would preclude safe splitting; a backup surgical plan in case of unexpected anatomical findings. Donor‐recipient matching parameters: graft‐to‐recipient weight ratio (GRWR) between 2% and 4% for pediatric recipients; graft volume to standard liver volume ratio ≥ 40%; adequate recipient abdominal cavity space and proper alignment for vascular anastomosis. It is important to note that the same selection criteria were applied to all donors, irrespective of age. However, because older donors (≥ 50 years) more frequently harbor comorbidities such as hypertension, diabetes, or subclinical atherosclerosis, the stringent application of these criteria resulted in a de facto stricter screening process for the elderly donor group.

### 2.4 Outcomes and Statistical Analysis

The primary outcomes were 1‐year patient survival and death‐censored graft survival. Graft loss was defined by retransplantation or documented graft failure; deaths with a functioning graft were censored in the graft‐survival analysis. Secondary outcomes included perioperative variables, postoperative liver biochemistry, and complications requiring surgical or interventional management. Liver function tests (ALT, AST, TBIL, INR) were measured on postoperative Days 1, 3, 7, and 14. Extended biochemical data beyond Day 14 were not collected per protocol; thus, results are limited to this 14‐day window.

Continuous variables are summarized as mean ± standard deviation or median (interquartile range), as appropriate; categorical variables are presented as number (percentage). Between‐group comparisons used analysis of variance, the Kruskal—Wallis test, the chi‐squared test, or Fisher's exact test, as appropriate. Survival was estimated using the Kaplan–Meier method and compared using the log‐rank test.

Cox proportional hazards models estimated associations with graft loss. Donor age was modeled per 10‐year increase with donor‐cluster robust standard errors. To assess the functional form of donor age, a parsimonious three‐knot restricted cubic spline was fitted at 7, 23, and 51 years, using 25 years as the reference. The proportional‐hazards assumption was examined using Schoenfeld residuals. Because only 10 graft‐loss events occurred, adjusted models were treated as exploratory and were deliberately limited.

An exploratory supplementary analysis compared ex situ and in situ splitting with respect to recipient and donor characteristics, cold ischemia time, postoperative AST and TBIL, vascular and biliary complications, and 1‐year patient and death‐censored graft survival. Cox models for splitting technique were additionally adjusted for donor age. Missingness was below 5% for all variables, and complete‐case analysis was used. Precision was evaluated using 95% confidence intervals rather than an observed‐effect post hoc power calculation. All tests were two‐sided, and *p* < 0.05 was considered statistically significant. Statistical analyses were performed using IBM SPSS Statistics for Windows, version 27.0 (IBM Corp., Armonk, NY, USA), and R version 4.5.2 (R Foundation for Statistical Computing, Vienna, Austria). Kaplan–Meier analyses, log‐rank tests, Cox proportional hazards models, and Schoenfeld residual diagnostics were performed using the survival package (version 3.8‐6). Restricted cubic spline basis functions were generated using the Hmisc package (version 5.2‐5), and the spline figure was produced using ggplot2 (version 4.0.3).

## 3 Results

### 3.1 Overall Characteristics

The cohort comprised 144 recipients: 21 (14.6%) in Group A, 103 (71.5%) in Group B, and 20 (13.9%) in Group C. Median recipient age was 11 months (IQR, 7–58 months), median body weight was 8.0 kg (IQR, 7.0–15.8 kg), and median body mass index was 16.5 kg/m^2^ (IQR, 15.1–18.2 kg/m^2^). Cholestatic disease was the leading indication (116/144, 80.6%). The 124 donors had a median age of 21.0 years (IQR, 14.0–37.8 years), and 94 (75.8%) were male. Median donor body weight was 60.0 kg (IQR 50.3–68.0 kg) and median donor BMI was 21.5 (IQR 19.6–23.9). The leading causes of donor death were trauma (55.6%) and cerebrovascular accident (37.3%). Hepatic steatosis was present in 28.2% of donors and was predominantly classified as absent or mild (< 30%). Baseline characteristics are shown in Tables 1 and 2.

**TABLE 1:** Baseline recipient characteristics stratified by donor age group.

Characteristic	Group A (*n* = 21)	Group B (*n* = 103)	Group C (*n* = 20)	*p*
Age (months)	10 (7.5, 43.5)	12 (7, 68)	8.5 (7.0, 26.25)	0.583
Sex (male/female)	15/6	60/43	10/10	0.364
Height (cm)	70.0 (67.0, 88.0)	69.0 (63.0, 109.0)	68.5 (65.25, 78.75)	0.924
Weight (kg)	8.0 (7.0, 13.25)	8.5 (7.0, 18.0)	8.0 (7.0, 9.19)	0.743
BMI (kg/m^2^)	16.87 ± 2.00	16.84 ± 2.78	16.20 ± 2.12	0.452
MELD/PELD score	17.52 ± 9.77	20.72 ± 10.30	18.95 ± 11.27	0.489
Diagnosis, *n* (%)				0.115^a^
Cholestatic disease	20 (95.2)	79 (76.7)	17 (85.0)	—
Metabolic liver disease	1 (4.8)	8 (7.8)	0 (0)	—
Liver tumor	0 (0)	3 (2.9)	1 (5.0)	—
Others	0 (0)	13 (12.6)	2 (10.0)	—
ABO compatibility, *n*				0.254
Compatible	18	93	20	—
Incompatible	3	10	0	—

*Note:* Values are mean ± SD, median (IQR), or *n* (%).

^a^ Fisher's exact test was used when expected counts were < 5.

**TABLE 2:** Baseline donor characteristics stratified by donor age group.

Characteristic	Group A (*n* = 11)	Group B (*n* = 93)	Group C (*n* = 20)	*p*
Age (years)	6.0 (5.0, 8.0)	25.0 (16.0, 33.0)	52.0 (51.0, 55.0)	—
Sex (male/female)	8/3	71/22	15/5	1.000^a^
Weight (kg)	22.14 ± 5.29	60.93 ± 10.23	66.90 ± 9.85	—
BMI (kg/m^2^)	16.85 ± 2.27	21.76 ± 2.91	23.78 ± 2.61	—
ICU stay (days)	2.0 (1.0–4.0)	2.0 (1.0–6.0)	3.0 (2.0–5.0)	0.412^a^
Cause of death, *n* (%)				0.006^a^
Trauma	4 (36.4)	58 (62.4)	7 (35.0)	—
Cerebrovascular accident	6 (54.5)	27 (29.0)	13 (65.0)	—
Hypoxic brain injury	0 (0)	7 (7.5)	0 (0)	—
Others	1 (9.1)	1 (1.1)	0 (0)	—
AST (U/L)	81.0 (48.0, 229.0)	50.0 (39.0, 101.5)	56.63 (34.60, 75.50)	0.170
ALT (U/L)	44.0 (18.0, 86.0)	40.0 (22.4, 72.0)	25.00 (15.03, 39.59)	0.177
Total bilirubin (μmol/L)	15.20 ± 9.93	19.73 ± 14.40	14.81 ± 6.86	0.298
Albumin (g/L)	35.69 ± 7.89	37.06 ± 7.35	34.73 ± 11.00	0.616
GGT (U/L)	61.36 ± 67.83	67.76 ± 74.62	51.85 ± 65.56	0.314
Serum sodium (mmol/L)	150.72 ± 18.76	150.88 ± 11.89	150.97 ± 10.87	0.889
Donor steatosis grade, *n* (%)				0.011^a^
None	11 (100)	68 (73.1)	11 (55.0)	
Mild (< 30%)	0 (0)	22 (23.7)	9 (45.0)	
Moderate (30%–60%)	0 (0)	3 (3.2)	0 (0)	
Type of steatosis (macrovesicular/microvesicular)	—	25/3^b^	8/1	0.011^a^

*Note:* Values are mean ± SD, median (IQR), or *n* (%).

^a^ Fisher's exact test was used when expected cell counts were < 5.

^b^ Three donors had both macrovesicular and microvesicular steatosis.

### 3.2 Patient and Graft Survival

**FIGURE 2:**
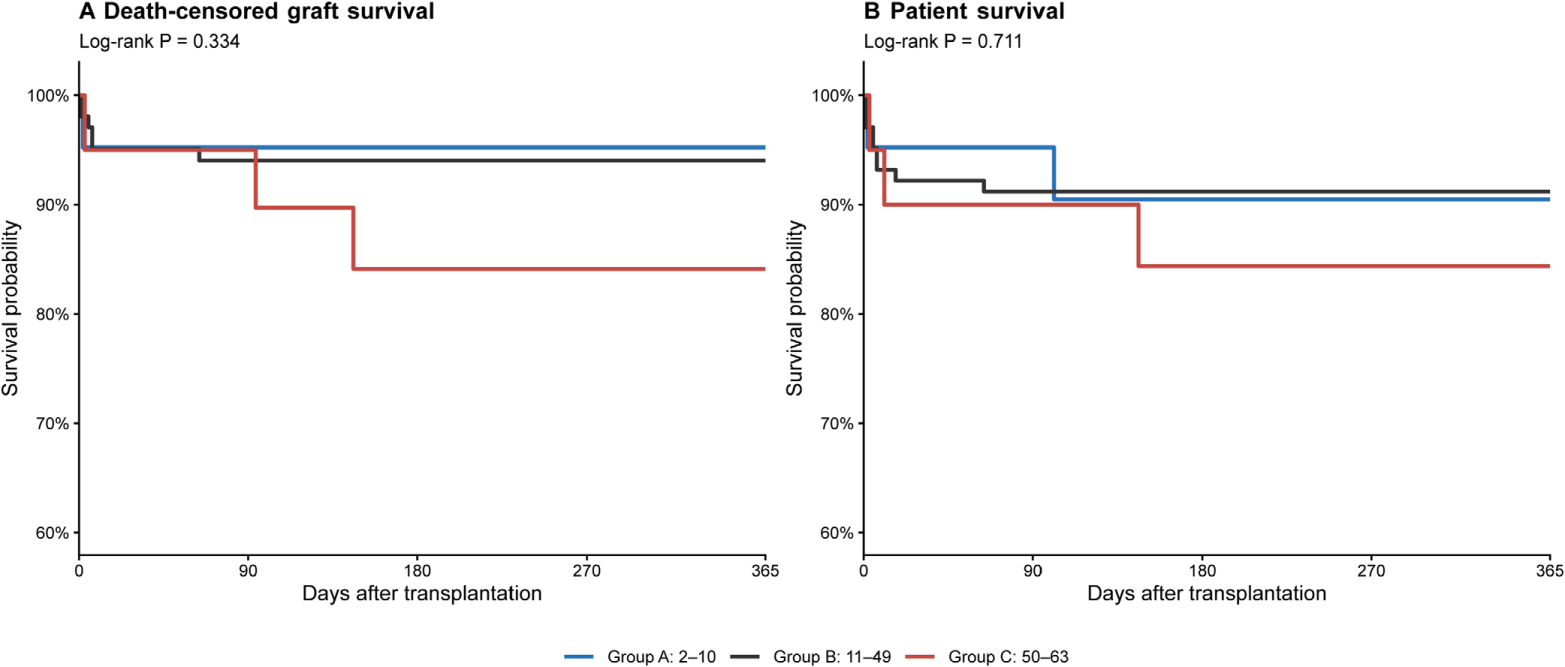
Kaplan–Meier estimates of 1‐year survival stratified by donor age. (A) Death‐censored graft survival (log‐rank *p* = 0.334). (B) Patient survival (log‐rank *p* = 0.711).

At 1 year, death‐censored graft survival was 95.2% (95% CI, 86.6%–100.0%) in Group A, 94.0% (95% CI, 89.5%–98.8%) in Group B, and 84.1% (95% CI, 69.1%–100.0%) in Group C (log‐rank *p* = 0.334). Corresponding patient survival was 90.5% (95% CI, 78.8%–100.0%), 91.2% (95% CI, 85.9%–96.9%), and 84.4% (95% CI, 69.5%–100.0%), respectively (*p* = 0.711) (Figure 2).

### 3.3 Causes of Graft Loss and Patient Death

Within the first postoperative year, graft loss occurred in 10 recipients (6.9%). Group A had one graft loss due to hepatic artery thrombosis. Group B had six graft losses attributed to portal vein thrombosis, hepatic artery thrombosis, primary nonfunction, or hemorrhagic shock related to hepatic malignancy. Group C had three graft losses attributed to portal vein thrombosis or chronic liver failure. Details are provided in Table 3.

**TABLE 3:** Graft loss within 1 year after transplantation.

Group	Cause of graft loss (*n*)	Recipient outcome
Group A (*n* = 1)	Hepatic artery thrombosis (1)	Died on postoperative Day 2
Group B (*n* = 6)	Portal vein thrombosis (3)	Died on postoperative Days 1, 5, and 64
	Hepatic artery thrombosis (1)	Underwent re‐transplantation on postoperative Day 2
	Primary non‐function (1)	Died on postoperative Day 1
	Hemorrhagic shock from liver malignancy (1)	Died on postoperative Day 7
Group C (*n* = 3)	Chronic liver failure (2)	Underwent re‐transplantation on postoperative Days 94 and 146; both died
	Portal vein thrombosis (1)	Died on postoperative Day 3

Fourteen recipients (9.7%) died within 1 year. Causes and timing of death are summarized in Table 4. Attribution analysis of deaths showed that all 3 deaths in Group C were graft‐related (100%), with 2 being late chronic liver failure; the proportions of graft‐related deaths in Group A and Group B were 50.0% and 55.6%, respectively, predominantly attributable to early vascular complications. The proportion of graft‐related deaths did not differ significantly among the three groups (*p* = 0.53). Overall, 71.4% (10/14) of deaths occurred within 30 days after surgery, with a median time to death of 6 days (range: 1–146 days). The proportion of early deaths in Group C (33.3%) was lower than that in Group A (50.0%) and Group B (88.9%), but the difference was not statistically significant (*p* = 0.131).

**TABLE 4:** Causes of recipient death within 1 year after transplantation.

Group	Cause of death (*n*)	Postoperative day of death
Group A (*n* = 2)	Hepatic artery thrombosis (1)	Day 2
	Pulmonary infection (1)	Day 101
Group B (*n* = 9)	Portal vein thrombosis (3)	Days 1, 5, and 64
	Cerebral hemorrhage with brain herniation (2)	Days 5 and 7
	Sepsis (1)	Day 1
	Primary non‐function (1)	Day 1
	Hemorrhagic shock from liver malignancy (1)	Day 7
	Pulmonary infection (1)	Day 17
Group C (*n* = 3)	Portal vein thrombosis (1)	Day 3
	Graft failure (chronic liver failure) (2)	Days 94 and 146

### 3.4 Graft Characteristics and Perioperative Outcomes

Graft type and splitting technique differed among age groups (both *p* < 0.001). All 21 recipients in Group A received ex situ split grafts, whereas in situ splitting was used in 46 Group B recipients (44.7%) and 8 Group C recipients (40.0%). The cold ischemia time was significantly longer in Group A than in Groups B and C (*p* = 0.026). Operative time was longest in Group C, but the intergroup difference did not reach statistical significance (*p* = 0.17). Two patients in the entire cohort with a GRWR ≤ 1.0% did not develop small‐for‐size syndrome (SFSS) or graft failure postoperatively. Anhepatic time, blood loss, operative time, and intensive care unit stay did not differ significantly (Table 5).

**TABLE 5:** Graft characteristics and perioperative outcomes.

Parameter	Group A (*n* = 21)	Group B (*n* = 103)	Group C (*n* = 20)	*p*
Graft type, *n* (%)				< 0.001^a^
LLS (including reduced)	7 (33.3)	82 (79.6)	18 (90.0)	
ERL	7 (33.3)	7 (6.8)	0 (0)	
LL	3 (14.3)	9 (8.7)	2 (10.0)	
RL	4 (19.0)	5 (4.9)	0 (0)	
Split technique, *n* (%)				< 0.001^a^
Ex situ	21 (100)	57 (55.3)	12 (60.0)	
In situ	0 (0)	46 (44.7)	8 (40.0)	
Graft weight (g)	541.76 ± 331.98	618.72 ± 372.80	632.65 ± 375.69	0.553
GRWR (%)	3.03 ± 1.35 median 2.96 (1.71–4.23), range 1.36–5.39	2.39 ± 1.08 median 2.06 (1.56–3.26), range 0.96–5.40	2.25 ± 1.19 median 2.07 (1.14–3.31), range 1.08–3.31	0.088
Anhepatic time (min)	54.29 ± 14.96	59.03 ± 12.02	56.65 ± 14.33	0.324
CIT (min)	492.89 ± 64.86	414.60 ± 119.25	448.69 ± 99.81	0.026
Intraoperative blood loss (mL)	200 (100–500)	200 (100–400)	200 (125–475)	0.774
Operative time (min)	451.81 ± 87.34	482.94 ± 112.56	497.60 ± 106.0	0.170
ICU stay (days)	4.0 (3.0–6.0)	4.0 (3.0–5.0)	4.0 (3.0–6.5)	0.499

Abbreviations: CIT, cold ischemia time; ERL, extended right lobe; GRWR, graft‐to‐recipient weight ratio; ICU, intensive care unit; LL, left lobe; LLS, left lateral segment; RL, right lobe.

^a^ Fisher's exact test was used when expected cell counts were < 5.

### 3.5 Postoperative Liver Function

**FIGURE 3:**
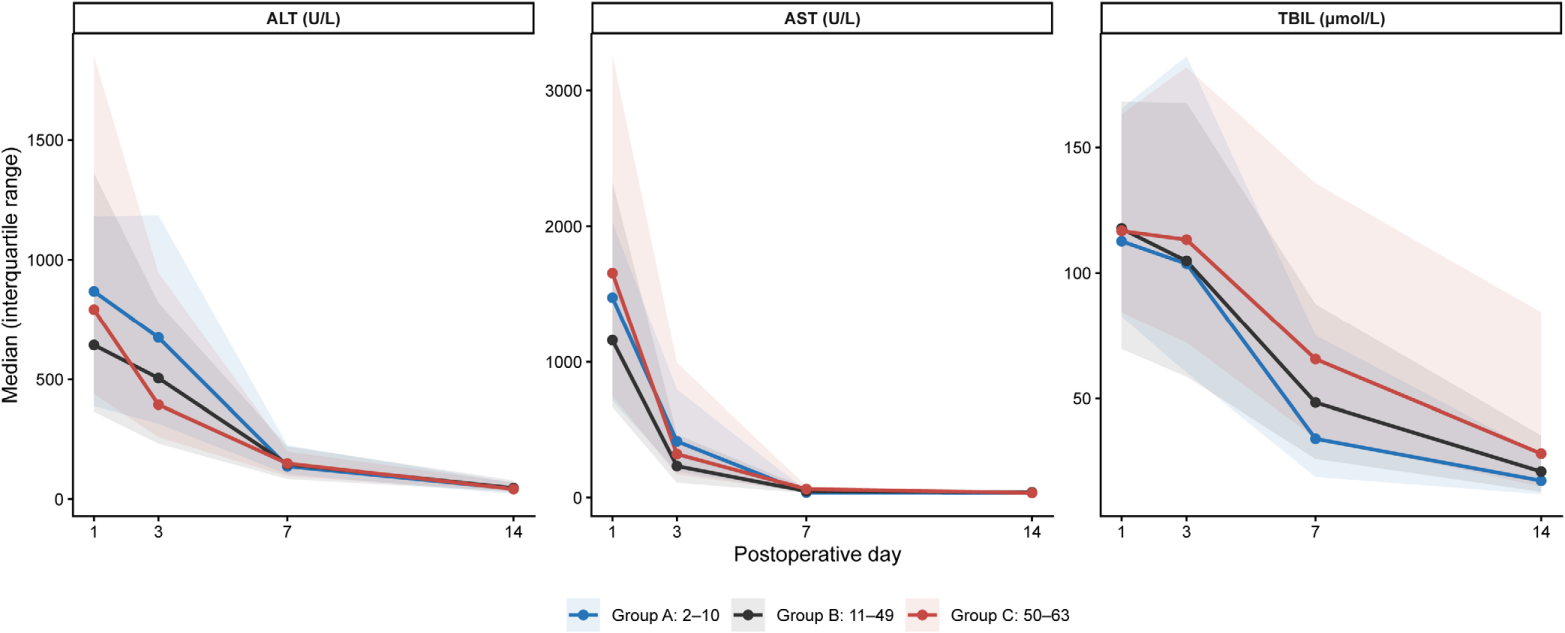
Postoperative liver‐function trajectories by donor‐age group. Lines show medians and shaded areas show interquartile ranges for ALT, AST, and total bilirubin (TBIL) on postoperative Days 1, 3, 7, and 14.

Liver enzymes, TBIL, and INR generally improved during the first 14 postoperative days. ALT and INR did not differ significantly among groups at any assessed time point. On postoperative Day 7, AST and TBIL were higher in Group C than in the younger groups (both *p* = 0.03), and TBIL showed a persistent trend toward slower decline on Day 14 (*p* = 0.05) (Figure 3).

### 3.6 Complications Within the First Postoperative Year

Only postoperative complications requiring surgical or interventional management (e.g., interventional thrombolysis, balloon angioplasty, laparotomy) were recorded. A total of 4, 20, and 7 vascular complication events were recorded in Groups A, B, and C, respectively. Event categories could overlap within the same recipient. With Group B as the reference, the odds ratio (OR) for Group C was 1.80 (95% CI, 0.87–3.74), and the intergroup difference was not statistically significant (*p* > 0.05). Subgroup analysis showed that the incidence of portal vein complications (thrombosis and stenosis) in Group C was 20.0%, which was higher than that in Groups A (9.5%) and B (9.7%); with Group B as the reference, the OR was 2.06 (95% CI, 0.70–6.07). Similarly, the incidence of hepatic vein stenosis in Group C was 15.0% (higher than that in Groups A and B), with an OR of 2.21 (95% CI, 0.59–8.29) compared with Group B. Hepatic artery thrombosis occurred in 4.8% (1/21) of patients in Group A and 2.9% (3/103) in Group B, but was not observed in Group C. The frequency of biliary complications did not differ significantly among the three groups (*p* = 0.614) (Table 6).

**TABLE 6:** Postoperative complications requiring surgical or interventional management.

Complication	Group A (*n* = 21)	Group B (*n* = 103)	Group C (*n* = 20)	*p*
Portal vein thrombosis	1 (4.8%)	2 (1.9%)	2 (10.0%)	0.164^a^
Portal vein stenosis	1 (4.8%)	8 (7.8%)	2 (10.0%)	0.123
Hepatic artery thrombosis	1 (4.8%)	3 (2.9%)	0 (0)	1.000
Hepatic vein stenosis	1 (4.8%)	7 (6.8%)	3 (15.0%)	0.538
Biliary complications	3 (14.3%)	8 (7.8%)	1 (5.0%)	0.614

Abbreviations: HAT, hepatic artery thrombosis; PVT, portal vein thrombosis.

^a^ Fisher's exact test was used when expected cell counts were < 5.

### 3.7 Continuous Donor‐Age and Cox Regression Analyses

When donor age was modeled continuously, each 10‐year increase was associated with a hazard ratio of 1.217 for death‐censored graft loss (95% CI, 0.823–1.800; *p* = 0.324) and 1.099 for patient death (95% CI, 0.796–1.516; *p* = 0.568). The restricted cubic spline showed no statistically significant departure from linearity (*p* for nonlinearity = 0.527; Figure S1). The proportional‐hazards diagnostic for donor age in the graft‐loss model was borderline (*p* = 0.049); an exploratory age‐by‐time interaction was not statistically significant (*p* = 0.066).

In the exploratory adjusted Cox model using donor‐cluster robust standard errors, donor age remained unassociated with graft loss (adjusted HR per 10‐year increase, 1.075; 95% CI, 0.801–1.441; *p* = 0.631). Postoperative PVT showed the largest association with graft loss (adjusted HR, 22.735; 95% CI, 5.966–86.644; *p* < 0.001). Operative time (adjusted HR per 1‐min increase, 1.009; 95% CI, 1.006–1.013; *p* < 0.001) and MELD/PELD score (adjusted HR per 1‐point increase, 1.098; 95% CI, 1.019–1.182; *p* = 0.014) were also associated with graft loss. Given that the model was based on only 10 graft‐loss events, these estimates are exploratory and should be interpreted cautiously (Table 7).

**TABLE 7:** Cox regression analyses of 1‐year death‐censored graft loss.

Variable	Unit/reference	Univariable HR (95% CI)	*p*	Exploratory adjusted HR (95% CI)	*p*
Donor age	Per 10‐year increase	1.217 (0.823–1.800)	0.324	1.075 (0.801–1.441)	0.631
Age group: Group A	Group B reference	0.792 (0.099–6.362)	0.826	—	—
Age group: Group C	Group B reference	2.545 (0.666–9.724)	0.172	—	—
MELD/PELD score	Per 1‐point increase	1.063 (0.998–1.133)	0.060	1.098 (1.019–1.182)	0.014
Operative time	Per 1‐min increase	1.007 (1.003–1.011)	< 0.001	1.009 (1.006–1.013)	< 0.001
Portal vein thrombosis	Yes vs. no	16.460 (4.181–64.806)	< 0.001	22.735 (5.966–86.644)	< 0.001

*Note:* HRs use donor‐cluster robust standard errors. The adjusted model included donor age (continuous), MELD/PELD score, operative time, and postoperative PVT. Because only 10 graft‐loss events occurred, adjusted results are exploratory.

Abbreviations: CI, confidence interval; HR, hazard ratio; PVT, portal vein thrombosis.

### 3.8 Exploratory Analysis by Splitting Technique

Ex situ splitting was used in 90 recipients and in situ splitting in 54. Compared with in situ splitting, ex situ splitting involved younger and smaller donors and substantially longer cold ischemia time (median, 495 vs. 308 min; *p* < 0.001). AST was higher after ex situ splitting on Days 1 and 3 but not on Days 7 or 14; TBIL did not differ significantly at any assessed time point. Vascular and biliary complications were similar. One‐year death‐censored graft survival was 93.1% after ex situ and 92.4% after in situ splitting (*p* = 0.863); patient survival was 88.8% and 92.4%, respectively (*p* = 0.490). After adjustment for donor age, splitting technique was not associated with graft loss or patient death (Table S1).

## 4 Discussion

Pediatric liver transplantation is constrained by a severe shortage of donor organs [19]. SLT expands the donor pool and shortens waiting times [1, 20], yet the impact of donor age on outcomes remains unclear [21]. The present study evaluated the effect of different donor age categories on 1‐year outcomes in a cohort of 144 pediatric SLT recipients.

Our data demonstrate that recipients of grafts from young donors (2–10 years), despite all grafts being procured by ex situ splitting and having significantly longer cold ischemia times than those from the reference group aged 11–49 years, achieved 1‐year patient and graft survival rates that were not statistically different from those of the standard age group. This finding contradicts the traditional view that prolonged cold ischemia time substantially increases the risk of graft loss [16, 22, 23]. A plausible underlying mechanism is that pediatric donor livers possess superior hepatocyte proliferative and regenerative capacity, and consequently tolerate cold ischemia–reperfusion injury far better than adult livers [24, 25], a property that may compensate for the potential damage inflicted by extended cold storage inherent to ex situ splitting. Nonetheless, the vascular complications associated with young donor grafts warrant vigilance: in the present study, the single case of graft loss in the young‐donor group resulted from hepatic artery thrombosis (HAT), underscoring that HAT remains the principal life‐threatening complication when using such grafts. Large multicenter studies have reported HAT incidences of 5.7%–8.4% [26], whereas the overall HAT rate in our cohort was 2.78% and the rate in the young‐donor group was 4.76%, both substantially lower than historical figures. This suggests that supermicrosurgical arterial anastomosis, standardized postoperative anticoagulation, and meticulous hemodynamic monitoring are key to the safe use of grafts from young donors [27]. Our institutional practice for preventing HAT includes the following elements: (1) routine hepatic artery reconstruction using 8‐0 or 9‐0 non‐absorbable sutures under the operating microscope with a continuous suture technique; (2) intraoperative ultrasonography after reperfusion and after abdominal closure to maintain a resistive index of 0.4–0.6; and (3) continuous intravenous infusion of unfractionated heparin at 20 U/kg/h immediately after surgery, followed by bridging to oral warfarin (target INR 1.5–2.5) for 3–6 months. This practice appears effective in preventing HAT.

In the present study, donor age ≥ 50 years was not found to significantly increase the risk of patient death or graft loss within the first year after pediatric SLT. This finding contrasts with data from the European Liver Transplant Registry, which identified donor age > 50 years as an independent risk factor for graft failure in pediatric SLT [16], and with the report by Olthoff et al. that donor age > 45 years significantly increases the risk of early allograft dysfunction (EAD) and graft loss [28]. This discrepancy is largely attributable to our center's stringent selection criteria for older donors: all enrolled donors were free of moderate‐to‐severe macrovesicular steatosis, had normal liver biochemistry, and had no evidence of underlying hepatic fibrosis. Nevertheless, recipients of older grafts showed significantly higher AST and TBIL levels on postoperative Day 7, with a trend toward delayed bilirubin clearance, which is consistent with the known age‐related decline in hepatocyte regenerative capacity and increased susceptibility to ischemia–reperfusion injury [29]. Notably, all three deaths in the older‐donor group were graft‐related, and two of these resulted from late chronic liver failure; previous studies have also suggested that a larger donor–recipient age difference is associated with elevated mortality risk [30]. These observations indicate that, although the short‐term safety of grafts from donors aged ≥ 50 years appears acceptable under strict selection protocols, the potential risk of late functional deterioration warrants continued long‐term surveillance.

The incidences of vascular and biliary complications requiring surgical or interventional management did not differ significantly among the three groups, which is consistent with the survival analysis. However, the overall vascular complication rate in the older‐donor group reached 35.0%, nearly double that in the 11–49 years reference group; the incidences of portal vein complications and hepatic vein stenosis were each more than twice those observed in the reference group. Although these intergroup differences were not statistically significant, the clinical trend should not be dismissed solely on the basis of a *p* > 0.05, given the small sample size of the older‐donor group.

From a pathophysiological perspective, older donors exhibit age‐related changes including decreased vascular wall elasticity, intimal atherosclerosis, and a higher prevalence of anatomical variations of the vascular branches [25, 31, 32]. These alterations may increase the technical difficulty of vascular anastomosis and elevate the risk of postoperative anastomotic stenosis and thrombosis, which is consistent with the trend toward higher vascular complication rates observed in the present study. Therefore, when grafts from older donors are used, meticulous attention must be paid to the quality of vascular anastomosis and to early screening for postoperative vascular complications.

In the exploratory multivariable Cox model using donor‐cluster robust standard errors, postoperative PVT showed the strongest association with graft loss (adjusted HR, 22.735; 95% CI, 5.966–86.644; *p* < 0.001). Among the five recipients who required surgical or interventional management for PVT, four experienced graft loss and died, highlighting the clinical severity of this complication. The incidence of PVT in this cohort was 3.47%, which is lower than the previously reported range of 5%–10% [33] but remains clinically significant. This finding is related to the fact that 80.6% of recipients had cholestatic liver disease, such as biliary atresia, a condition frequently accompanied by portal vein hypoplasia and fibrotic remodeling [34]. In addition, the small caliber of the pediatric portal vein and the common size mismatch with adult split grafts together contribute to the elevated risk of PVT. Accordingly, a comprehensive, protocol‐driven perioperative PVT prevention and management strategy for pediatric SLT should be established: preoperatively, routine portal vein imaging and thrombus screening should be performed, with preoperative anticoagulation for those with positive findings [35]; intraoperatively, donor–recipient matching should be optimized to avoid vessel kinking or excessive tension, microsurgical anastomosis should be performed by experienced surgeons in high‐risk cases, and thrombectomy may be considered for de novo PVT [36]; postoperatively, thromboelastography‐guided individualized anticoagulation is recommended [37, 38]. Our institutional postoperative anticoagulation protocol consists of continuous intravenous infusion of unfractionated heparin sodium at 20 U/kg/h, initiated immediately after transplantation. Oral warfarin is introduced at approximately 1 week after transplantation, with bridging anticoagulation until the target international normalized ratio of 1.5–2.5 is achieved. Warfarin therapy is subsequently continued for 3–6 months. This protocol is intended to reduce the risk of postoperative portal vein thrombosis.

Furthermore, the GRWR is another important factor influencing outcomes after SLT. Most studies recommend a minimum GRWR of 0.8%–1.0% for adult recipients of deceased donor grafts [14, 15, 39]. Because split grafts are often exposed to prolonged cold ischemia time and to the hemodynamic insults associated with brain death, it has been traditionally held that SLT recipients may require a higher GRWR threshold [40]. In the present study, however, two patients with a GRWR ≤ 1.0% developed neither small‐for‐size syndrome (SFSS) nor graft failure, and both had favorable short‐term outcomes. This finding provides evidence to support further expansion of the donor pool for pediatric SLT.

This study has several limitations. First, its retrospective design is inherently subject to selection bias and confounding, and the small sample sizes of the young‐donor and older‐donor groups may limit the precision of the risk estimates for extreme donor ages. Second, this is a single‐center experience in which all procedures were performed by a highly experienced transplant team; therefore, the generalizability of the findings should be interpreted with caution. Moreover, the follow‐up period was limited to 1 year post‐transplantation, and only the short‐term impact of donor age on pediatric SLT outcomes was analyzed. Finally, differences in immunosuppressive regimens were not taken into account; variations in drug choice and in the achievement of target trough levels among individual patients may have influenced outcomes [41, 42]. These aspects will be addressed in future studies.

## 5 Conclusion

One‐year patient and death‐censored graft survival did not differ significantly by donor age. Carefully selected young and older donors may be considered in experienced centers, but small extreme‐age groups and wide confidence intervals warrant caution, particularly regarding vascular complications and delayed functional recovery in older grafts.

## Funding

This work was supported by the Guangzhou Regional Clinical High‐level, Major and Characteristic Technology Program (2026–2028) (grant no. 2026P‐GX007).

## Conflicts of Interest

The authors declare no conflicts of interest. The funders had no role in study design; data collection, analysis, or interpretation; manuscript preparation; or the decision to submit the work.

## Supporting information


**Table S1:** Exploratory comparison of ex situ and in situ splitting. Continuous data are median (IQR); categorical data are *n* (%). Survival was estimated by Kaplan–Meier methods. Donor‐age‐adjusted Cox models yielded HR 0.983 (95% CI, 0.239–4.043; *p* = 0.981) for graft loss and HR 0.616 (95% CI, 0.176–2.151; *p* = 0.447) for patient death for in situ versus ex situ splitting.
**Figure S1:** Restricted cubic spline for donor age and 1‐year death‐censored graft loss. The model used knots at 7, 23, and 51 years and a reference age of 25 years. Shading indicates the 95% confidence interval; P for nonlinearity = 0.527.

## Data Availability

The data that support the findings of this study are available on request from the corresponding author. The data are not publicly available due to privacy or ethical restrictions.
