# Supplementary material for: Impact of Donor Age on Short‐Term Outcomes After Pediatric Split Liver Transplantation
Source: Pediatr Transplant. 2026 Aug 27;30(9):e70444. doi: 10.1111/petr.70444 (PMC13519970; doi:10.1111/petr.70444)
Supplement: Supplementary file 1 [file PETR-30-e70444-s001.docx]

| Variable | Ex situ (n=90) | In situ (n=54) | P |
| --- | --- | --- | --- |
| Recipient age (months) | 9.5 (7.0–36.2) | 17.0 (7.2–91.0) | 0.086 |
| Recipient weight (kg) | 8.0 (6.8–13.5) | 9.1 (7.0–20.4) | 0.188 |
| Recipient BMI (kg/m²) | 16.6 (15.4–18.1) | 16.4 (14.6–18.2) | 0.192 |
| MELD/PELD score | 20.0 (13.2–28.8) | 17.5 (12.0–27.0) | 0.190 |
| Donor age (years) | 19.5 (12.2–35.8) | 26.5 (16.0–41.8) | 0.009 |
| Donor weight (kg) | 55.0 (35.2–65.0) | 60.0 (58.0–65.8) | 0.017 |
| Donor BMI (kg/m²) | 20.5 (17.8–23.0) | 22.0 (20.7–23.9) | 0.001 |
| Cold ischemia time (min) | 495.0 (450.0–540.0) | 308.0 (268.5–393.0) | <0.001 |
| AST day 1 (U/L) | 1481.0 (763.2–2880.2) | 939.0 (463.0–1832.0) | 0.013 |
| AST day 3 (U/L) | 329.0 (171.5–765.0) | 179.0 (92.0–350.0) | 0.002 |
| AST day 7 (U/L) | 48.0 (35.2–81.0) | 46.5 (34.0–63.8) | 0.235 |
| AST day 14 (U/L) | 39.0 (28.0–54.0) | 36.5 (26.2–55.5) | 0.952 |
| TBIL day 1 (μmol/L) | 116.0 (71.3–163.0) | 137.7 (76.0–183.4) | 0.280 |
| TBIL day 3 (μmol/L) | 110.4 (69.1–178.7) | 89.2 (41.4–152.3) | 0.067 |
| TBIL day 7 (μmol/L) | 52.3 (27.4–88.3) | 43.9 (21.3–108.5) | 0.506 |
| TBIL day 14 (μmol/L) | 19.2 (13.4–36.0) | 22.3 (12.8–33.6) | 0.885 |
| Male recipient, n (%) | 56 (62.2%) | 29 (53.7%) | 0.382 |
| Cholestatic disease, n (%) | 77 (85.6%) | 39 (72.2%) | 0.080 |
| ABO incompatible, n (%) | 10(11.1%) | 3(5.5%) | 0.252 |
| Male donor, n (%) | 70 (77.8%) | 37 (68.5%) | 0.241 |
| Donation after brain death, n (%) | 79 (87.8%) | 54 (100.0%) | 0.007 |
| Traumatic donor death, n (%) | 48 (53.3%) | 31 (57.4%) | 0.730 |
| Any donor steatosis, n (%) | 17 (18.9%) | 19 (35.2%) | 0.046 |
| Portal vein thrombosis, n (%) | 3 (3.3%) | 2 (3.7%) | 1.000 |
| Portal vein stenosis, n (%) | 9 (10.0%) | 2 (3.7%) | 0.132 |
| Hepatic artery thrombosis, n (%) | 3 (3.3%) | 1 (1.9%) | 1.000 |
| Hepatic vein complication, n (%) | 6 (6.7%) | 5 (9.3%) | 0.747 |
| Biliary complication, n (%) | 8 (8.9%) | 4 (7.4%) | 1.000 |
| Any vascular complication, n (%) | 18 (20.0%) | 10 (18.5%) | 1.000 |
| Death-censored graft survival at 1 year | 93.1% (95% CI 87.9–98.6) | 92.4% (95% CI 85.5–99.8) | 0.863 |
| Patient survival at 1 year | 88.8% (95% CI 82.5–95.6) | 92.4% (95% CI 85.5–99.8) | 0.490 |

Table S1. Exploratory comparison of ex situ and in situ splitting.

Continuous data are median (IQR); categorical data are n (%). Survival was estimated by Kaplan–Meier methods. Donor-age-adjusted Cox models yielded HR 0.983 (95% CI, 0.239–4.043; P=0.981) for graft loss and HR 0.616 (95% CI, 0.176–2.151; P=0.447) for patient death for in situ versus ex situ splitting.

Figure S1. Restricted cubic spline for donor age and 1-year death-censored graft loss. The model used knots at 7, 23, and 51 years and a reference age of 25 years. Shading indicates the 95% confidence interval; P for nonlinearity=0.527.
